# CONNECTING THROUGH TECHNOLOGY: EXAMINING THE RELATIONSHIP BETWEEN SOCIAL NETWORKING SITES AND MENTAL HEALTH

**DOI:** 10.1093/geroni/igad104.2721

**Published:** 2023-12-21

**Authors:** Joseph Svec, Yeon Ji Ryou, Jeong Eun Lee

**Affiliations:** Saint Joseph’s University, New York City, New York, United States; Iowa State University, Ames, Iowa, United States; Iowa State University, Ames, Iowa, United States

## Abstract

Social isolation and loneliness among older adults correspond with numerous mental and physical health consequences including but not limited to depression, anxiety, declines in cognitive functioning, and poor health behaviors. More recent research highlights the potential benefits of digital-social communication technology through social networking sites (SNS), communicative technology, and computer or tablet use (Khosravi et al. 2016; Jutai and Tuazon 2022; Neil-Sztramko et al. 2020). However, the efficacy of technology on mental and physical well-being remains obscure, particularly given the often interchangeability of isolation and loneliness concepts. In this research, we examine the extent to which conditions of isolation and feelings of loneliness correspond with depression among older adults. Using data from two waves of Health and Retirement Study (HRS) data in 2018 and 2020 (N = 8,994), we conduct a series of linear regressions of depression scales on both isolation and loneliness as well as their interaction with the use of social technology in communication. Preliminary results suggest that that the use of virtual communications by social networking sites (SNS) moderates the associations between isolation and depression levels, but no such effect is observed in terms of self-perception of loneliness. Moreover, the magnitude of SNS-Isolation and depression linkages differs significantly for family and friends. This research contributes to the growing body of literature on the intersection of technology and psychological well-being among isolated older adults, highlighting the both the potential benefits and limitations of virtual communication for improving health outcomes in older adults.

